# Monitoring the Implementation of Tobacco Cessation Support Tools: Using Novel Electronic Health Record Activity Metrics

**DOI:** 10.2196/43097

**Published:** 2023-03-02

**Authors:** Jinying Chen, Sarah L Cutrona, Ajay Dharod, Stephanie C Bunch, Kristie L Foley, Brian Ostasiewski, Erica R Hale, Aaron Bridges, Adam Moses, Eric C Donny, Erin L Sutfin, Thomas K Houston, iDAPT Implementation Science Center for Cancer Control

**Affiliations:** 1 iDAPT Implementation Science Center for Cancer Control Wake Forest University School of Medicine Winston-Salem, NC United States; 2 Department of Internal Medicine Wake Forest University School of Medicine Winston-Salem, NC United States; 3 Department of Population and Quantitative Health Sciences University of Massachusetts Chan Medical School Worcester, MA United States; 4 Department of Preventive Medicine and Epidemiology Boston University Chobanian & Avedisian School of Medicine Boston, MA United States; 5 Department of Implementation Science Division of Public Health Sciences Wake Forest University School of Medicine Winston-Salem, NC United States; 6 Wake Forest Center for Healthcare Innovation Winston-Salem, NC United States; 7 Wake Forest Center for Biomedical Informatics Winston-Salem, NC United States; 8 Center for Health Analytics, Media, and Policy RTI International Research Triangle Park, NC United States; 9 Clinical & Translational Science Institute Wake Forest University School of Medicine Winston-Salem, NC United States; 10 Department of Physiology and Pharmacology Wake Forest University School of Medicine Winston-Salem, NC United States; 11 Department of Social Sciences and Health Policy Wake Forest University School of Medicine Winston-Salem, NC United States; 12 Wake Forest University School of Medicine Winston-Salem, NC United States

**Keywords:** medical informatics, electronic health records, EHR metrics, alerts, alert burden, tobacco cessation, monitoring, clinical decision support, implementation science, smoking cessation, decision tool

## Abstract

**Background:**

Clinical decision support (CDS) tools in electronic health records (EHRs) are often used as core strategies to support quality improvement programs in the clinical setting. Monitoring the impact (intended and unintended) of these tools is crucial for program evaluation and adaptation. Existing approaches for monitoring typically rely on health care providers’ self-reports or direct observation of clinical workflows, which require substantial data collection efforts and are prone to reporting bias.

**Objective:**

This study aims to develop a novel monitoring method leveraging EHR activity data and demonstrate its use in monitoring the CDS tools implemented by a tobacco cessation program sponsored by the National Cancer Institute’s Cancer Center Cessation Initiative (C3I).

**Methods:**

We developed EHR-based metrics to monitor the implementation of two CDS tools: (1) a screening alert reminding clinic staff to complete the smoking assessment and (2) a support alert prompting health care providers to discuss support and treatment options, including referral to a cessation clinic. Using EHR activity data, we measured the completion (encounter-level alert completion rate) and burden (the number of times an alert was fired before completion and time spent handling the alert) of the CDS tools. We report metrics tracked for 12 months post implementation, comparing 7 cancer clinics (2 clinics implemented the screening alert and 5 implemented both alerts) within a C3I center, and identify areas to improve alert design and adoption.

**Results:**

The screening alert fired in 5121 encounters during the 12 months post implementation. The encounter-level alert completion rate (clinic staff acknowledged completion of screening in EHR: 0.55; clinic staff completed EHR documentation of screening results: 0.32) remained stable over time but varied considerably across clinics. The support alert fired in 1074 encounters during the 12 months. Providers acted upon (ie, not postponed) the support alert in 87.3% (n=938) of encounters, identified a patient ready to quit in 12% (n=129) of encounters, and ordered a referral to the cessation clinic in 2% (n=22) of encounters. With respect to alert burden, on average, both alerts fired over 2 times (screening alert: 2.7; support alert: 2.1) before completion; time spent postponing the screening alert was similar to completing (52 vs 53 seconds) the alert, and time spent postponing the support alert was more than completing (67 vs 50 seconds) the alert per encounter. These findings inform four areas where the alert design and use can be improved: (1) improving alert adoption and completion through local adaptation, (2) improving support alert efficacy by additional strategies including training in provider-patient communication, (3) improving the accuracy of tracking for alert completion, and (4) balancing alert efficacy with the burden.

**Conclusions:**

EHR activity metrics were able to monitor the success and burden of tobacco cessation alerts, allowing for a more nuanced understanding of potential trade-offs associated with alert implementation. These metrics can be used to guide implementation adaptation and are scalable across diverse settings.

## Introduction

### Background

Provider-facing computerized clinical decision support (CDS) tools in electronic health records (EHRs) are common digital health interventions supporting health care quality improvement programs [1-6]. Monitoring (ie, continual evaluation) of the impact of these tools is important for program evaluation and may ultimately contribute to implementation success [7,8]. Approaches for evaluating CDS tools largely rely on surveys, qualitative interviews, and data collected through direct observation or audio/video recording [9-12]. These approaches require substantial human effort (from implementation staff and clinical teams) for data collection. Automated methods leveraging EHR activity data offer a promising solution to reduce the data collection burden, but research on these methods is still in the earliest stage.

This study aimed to develop automatic metrics to monitor the implementation of EHR-embedded CDS tools and demonstrate their use within the context of a smoking cessation program sponsored by a National Cancer Institute (NCI)–designated cancer center.

### Tobacco Control Programs in NCI Cancer Centers

Tobacco use increases the risk of cancer and leads to poor prognosis after cancer diagnosis [13-16]. Clinical practice guidelines recommend routine screening for tobacco use and referral to evidence-based cessation interventions in patients with cancer [17,18], but this practice is underused [19]. To address this practice gap, the NCI’s Beau Biden Cancer Moonshot program launched the Cancer Center Cessation Initiative (C3I) in 2017 to provide funding to NCI-designated cancer centers to implement or enhance their tobacco treatment services [20].

Electronic alerts (e-alerts) are common CDS tools in EHRs, promoting adherence to practice guidelines [2-5,21,22], including tobacco screening and treatment at the point of care [23-25]. This strategy has been adopted by some C3I-funded cancer centers [26,27]. However, effective implementation of alerts into the clinical workflow is nontrivial [28-32]. Monitoring of provider responses to newly implemented alerts can identify barriers to adoption and the burden imposed by the alerts.

### Study Objectives

We developed and applied EHR activity metrics to answer three questions. (1) Did the alert completion rate change over time or vary across clinics? (2) What was the burden introduced by the alerts? (3) What factors were associated with variation in alert completion? Our research questions were motivated by three factors. First, sustainability (eg, sustained use and completion of the alerts) is a key construct of implementation outcomes [8] and should be monitored over time. Second, monitoring variations in alert completion across clinics can support the adaptation of alert implementation to the local context. Third, alerts could add a “burden” on providers [30-33], which should be evaluated.

## Methods

### Study Design

**Figure 1:**
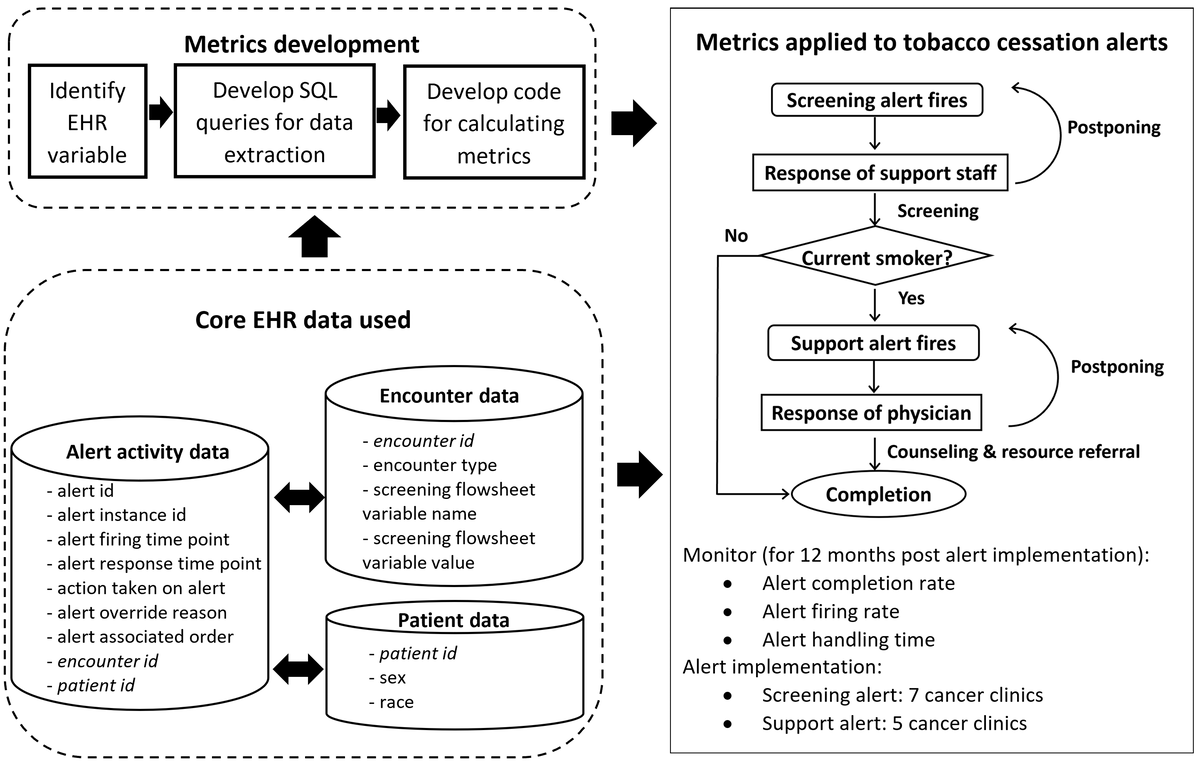
Study overview. The support alert would fire only if the screening result was positive (ie, patient being a current smoker) and answers to both Smoking Screener questions (Q1: “When did you last smoke (even 1 or 2 puffs)?”; Q2: “Quitting smoking could help improve your health. Are you interested in quitting?”) were documented (see Multimedia Appendix 1, step B1). EHR: electronic health record.

We developed and applied new EHR activity metrics to monitor the tobacco cessation tools (two Best Practice Advisory [BPA] alerts) implemented in cancer clinics for 12 months (Figure 1).

### Ethics Approval

The study was approved by the Wake Forest School of Medicine Institutional Review Board (IRB00066841). Deidentified EHR data were used, with informed consent for data access waved by the institutional review board.

### Digital Health Intervention

The CDS tools were two conditional sequential alerts integrated into the Epic EHR, a commercial cloud-based EHR system (Figure 1; detailed in Multimedia Appendix 1): (1) a screening alert to remind clinic staff to complete tobacco screening, triggered if “current smoker” or “unknown smoking status” was previously documented in the EHR, and (2) a support alert to prompt the clinical provider to discuss support and referral to a tobacco cessation clinic, triggered if the screening result was positive and answers to both Smoking Screener questions were answered (see note for Figure 1). Each alert had two modalities: (1) interruptive (triggered when the patient chart was opened; if postponed, presenting again after 10 minutes or when the patient chart was reopened) and (2) noninterruptive (in the general BPA section of the EHR).

### Implementation Context

The Tobacco Control Center of Excellence (TCCOE) at the Wake Forest Baptist Comprehensive Cancer Center implemented the alerts in the Epic EHR system used by 7 cancer clinics (medical oncology: n=3; radiation oncology: n=3; cancer survivorship: n=1) in the Atrium Health Wake Forest Baptist Comprehensive Cancer Center in 2019 and 2020. The alerts were integrated into the Epic EHR as BPAs, a form of CDS in the EHR that reminds providers to attend to important tasks [4]. The implementation team from the TCCOE worked with the hospital information technology team on implementing the alerts. The alerts were customized by using rule-based logic (eg, rules on who will receive the alerts and when to fire the alerts; detailed in Multimedia Appendix 1). All 7 clinics implemented the screening alert; 5 implemented the support alert. Training was provided to clinic staff and providers (1-month weekly before or in the first month of implementation and monthly check-in after alert implementation). Some clinics used extensive support from patient navigators and tobacco treatment specialists to complete screening documentation and referral to the cessation clinic.

### Evaluation

#### Metrics Development and Automation

Metrics development took three steps: (1) identifying relevant EHR variables, (2) developing SQL queries to extract variables from the EHR database, and (3) developing computer code to calculate the metrics. We used EHR data associated with 2 clinics to develop and test the metrics. A team of experts in health informatics and implementation science, EHR specialists, and physicians participated in the metrics development.

Using the computer code we developed, EHR data extraction takes about 10 minutes, and the calculation of each metric takes tens of seconds. This speed is adequate for monitoring CDS tools used by implementation programs. Full automation of these metrics is possible after their integration into the EHR.

#### EHR Variables Used to Derive the Metrics

We extracted alert activity data from event log files of the Epic EHR system. The variables used to develop the metrics included *alert id*, *alert instance id*, *alert name* (eg, a tobacco screening alert), timestamps corresponding to alert firing and provider responding (called *alert firing time point* and *alert response time point* for convenience), *alert triggering condition* (eg, triggered by opening the patient chart), *subsequent actions taken* (eg, acknowledge/override warning), *alert override reason*, and *alert-associated signed orders*.

Each *alert id* is associated with a unique *encounter id* and a *patient id*. An *alert id* corresponds to multiple *alert instance ids* if the alert is fired again after being postponed. *Alert triggering condition* was used to distinguish interruptive alerts from noninterruptive ones.

We used *subsequent actions taken*, *alert override reason*, and *alert-associated signed orders* to identify providers’ actions on the alerts. When the clinic staff completed or postponed the screening alert, *subsequent actions taken* recorded a value “acknowledge/override warning,” and *alert override* *reason* recorded whether the staff acknowledged screening completion (ie, hit the button “Documented in Flowsheet,” step A in Figure A1-1, Multimedia Appendix 1), postponed the alert (hit “Defer”), or determined that the patient was inappropriate for screening (hit “Not appropriate”). For the support alert, *subsequent actions taken* recorded a value “acknowledge/override warning” when the provider hit the buttons under “acknowledge reason,” and *alert override reason* recorded the provider’s actions (eg, discussed or not discussed with patients) and patient’s readiness to quit (Figure A1-2, Multimedia Appendix 1). *Alert-associated signed orders* recorded whether the provider placed an order for a referral to the cessation clinic.

In addition, we used two encounter-level variables, *flowsheet name* and *flowsheet value*, to determine whether the clinic staff documented screening results (ie, answers to Q1-Q3 in step B1 in Figure A1-1, Multimedia Appendix 1) in the EHR.

#### Metrics

We defined three metrics to measure alert completion and burden (Multimedia Appendix 2).

The *alert completion rate* was defined as the number of encounters where a provider completed alert-prompted actions divided by the number of encounters where the alert fired. We defined screening alert completion by either staff’s acknowledging completion of screening or completion of EHR documentation of screening results. We defined support alert completion at two levels: (1) discussing with patients and assessing patient readiness to quit (“discussion”) or (2) referring patients to the on-site tobacco cessation clinic (“referral”).

We measured the burden of interruptive alerts by two metrics: *alert firing rate* and *alert handling time*. We focused on interruptive alerts because they were more likely to add a “burden” on providers [30-33]. We defined *alert firing rate* as the number of times the alert fired during a specific period divided by the number of times the alert was completed during that period. We calculated the average time providers spent completing an alert per encounter, using encounters in which the alert was completed; similarly, we calculated the average time spent postponing alerts per encounter using encounters in which the alert was postponed at least once.

### Data Collection

For each clinic, we collected EHR data about clinic characteristics, patient characteristics, and EHR activities related to tobacco cessation alerts. We collected EHR alert activity data as described previously. Each instance of an alert was linked to a specific patient encounter and the patient’s demographic information (sex and race), using encounter and patient IDs.

### Data Analysis

We summarized clinic and patient characteristics for each clinic. We then used EHR activity metrics to address the research questions related to alert completion and burden. Statistical analyses were conducted using STATA/MP 15.1 (StataCorp LLC) [34].

We measured the overall and per-clinic alert completion rates for the screening alert during every 3-month period across 12 months post alert implementation. The 12-month postimplementation period was specified for each clinic. We measured the support alert completion rate at two levels: “discussion” and “referral.”

We measured the alert firing rate and handling time of interruptive screening alerts and support alerts to assess the burden of interruptive alerts.

### Factors Associated With Alert Completion

As a secondary analysis, we examined the distribution of alert completion over patients’ demographics (sex and race) and encounter types.

Three physicians reviewed all encounter types and selected “relevant encounter” types as those in which screening for smoking status was an appropriate part of routine care (Multimedia Appendix 3).

## Results

### Clinic Characteristics

The clinics varied in the number of encounters (from n=1464 to n=110,553) and patients (from n=328 to n=9410) during 12 months post alert implementation (Table 1). The typical structure of these clinics was for nurses to support multiple providers across multiple days.

**Table 1:** Clinic and patient characteristics during the 12 months after implementing tobacco cessation alerts.

				Medical oncology^a^						Radiation oncology^b^					Cancer survivorship (S^c^)
				M1^d^		M2		M3	R1^d^		R2	R3			
**Clinic characteristics**															
	Service area		Urban		Rural		Urban		Urban		Rural	Urban	Urban		
	Staffing, n^e^		5-10		10-20		100-110		10-20		5-10	20-30	10-20		
	Encounters, n		30,727		9102		110,553		4670		2769	21,362	1464		
	Patients, n		4688		1193		9410		1059		328	3196	623		
**Patient characteristics**															
	Age (years), mean (SD)		64 (14)		65 (13)		61 (15)		66 (11)		67 (11)	64 (14)	59 (19)		
	**Sex, n (%)^f^**														
		Female	3122 (66.6)		766 (64.2)		4956 (52.7)		603 (56.9)		156 (47.6)	1479 (46.3)	327 (52.5)		
		Male	1566 (33.4)		427 (35.8)		4454 (47.3)		456 (43.1)		172 (52.4)	1716 (53.7)	296 (47.5)		
	**Race, n (%)^f^**														
		African American	1070 (22.8)		160 (13.4)		1731 (18.4)		223 (21.1)		50 (15.2)	512 (16.0)	98 (15.7)		
		White	3350 (71.5)		988 (82.8)		7227 (76.8)		780 (73.7)		263 (80.2)	2546 (79.7)	500 (80.3)		
		Other^g^	259 (5.5)		45 (3.8)		436 (4.6)		53 (5.0)		14 (4.3)	136 (4.3)	24 (3.9)		
	**Hispanic or Latino, n (%)^f^**														
		Yes	114 (2.4)		23 (1.9)		349 (3.7)		19 (1.8)		8 (2.4)	85 (2.7)	20 (3.2)		
		No	4545 (96.9)		1170 (98.1)		9042 (96.1)		1034 (97.6)		320 (97.6)	3104 (97.1)	603 (96.8)		
	**Insurance, n (%)**														
		Medicare	2721 (58.0)		775 (65.0)		4961 (52.7)		646 (61.0)		205 (62.5)	1699 (53.2)	334 (53.6)		
		Medicaid	243 (5.2)		81 (6.8)		598 (6.4)		60 (5.7)		19 (5.8)	190 (5.9)	27 (4.3)		
		Other insurance	1659 (35.4)		305 (25.6)		3537 (37.6)		334 (31.5)		99 (30.2)	1226 (38.4)	253 (40.6)		
		No insurance	65 (1.4)		32 (2.7)		266 (2.8)		19 (1.8)		5 (1.5)	77 (2.4)	9 (1.4)		
	Smoking rate, n/N (%)^h^		590/4606 (12.8)		198/1150 (17.2)		1006/9230 (10.9)		174/1051 (16.6)		58/325 (17.8)	435/3155 (13.8)	45/506 (8.9)		

^a^M1-M3: medical oncology clinics 1-3.

^b^R1-R3: radiation oncology clinics 1-3.

^c^S: cancer survivorship clinic.

^d^M1 and R1 implemented only the screening alert.

^e^The approximate number of clinic team members (physicians, advanced practice practitioners, nurses, and other clinical staff) in a clinic. The number is not precise due to staff turnover and the hiring of temporary staff.

^f^Some clinics have a small percentage of patients missing information on sex (0.03% missing for R3; complete for other clinics), race (complete for M2; less than 0.3% missing for other clinics), and ethnicity (1% missing for M1, 0.6% missing for R1, 0.2% missing for M3 and R3; complete for other clinics).

^g^Other: American Indian or Alaska Native, Asian, Native Hawaiian or Other Pacific Islander, Latin American or Hispanic, and other.

^h^The percent of patients who were active smokers during 12 months post alert implementation. The denominator is the number of patients who had their smoking status documented in the electronic health record.

### Patient Characteristics

The patients seen by the cancer survivorship clinic were 5-8 years older than patients seen by other clinics (mean age for each clinic 59-67; Table 1). Most patients were non-Hispanic White and were beneficiaries of Medicare. The smoking rate ranged between 8.9% (n=45 among 506 patients who had smoking status documented in the EHR; cancer survivorship clinic) and 17.8% (58/325; radiology oncology clinic 2).

### Alert Completion Rate

**Figure 2:**
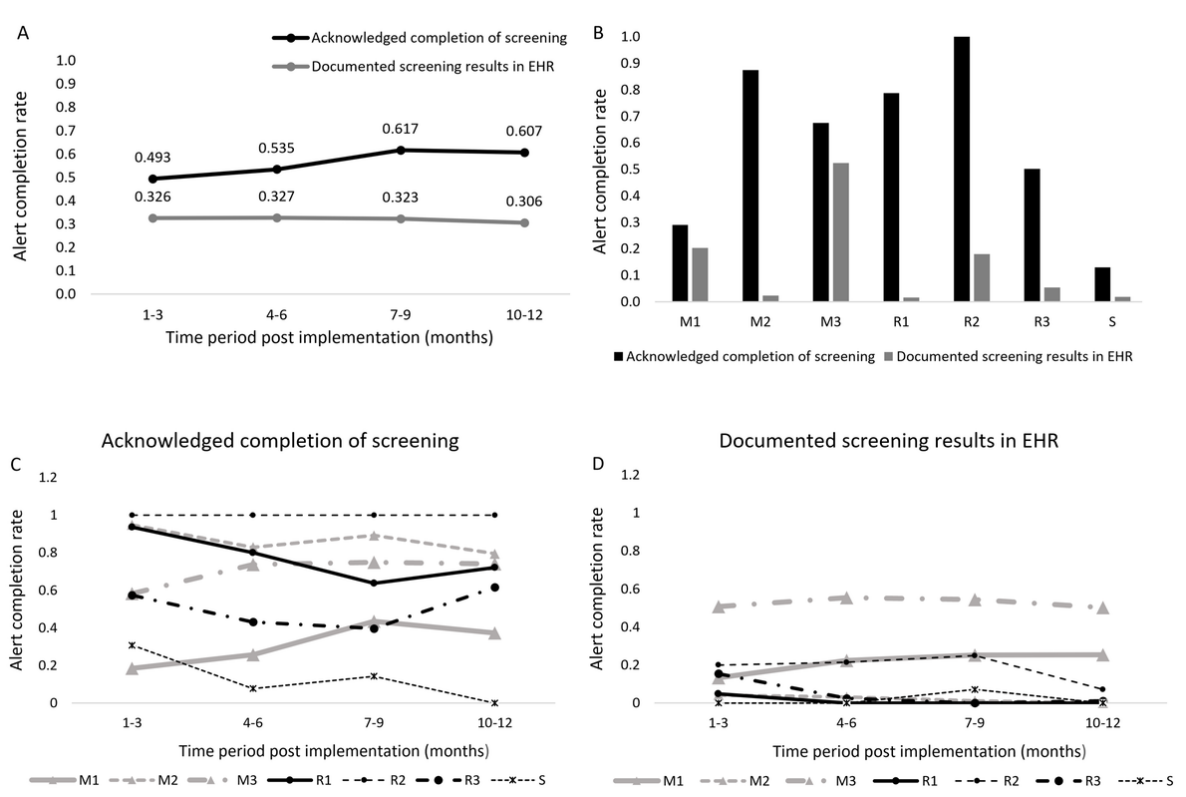
Completion rate of tobacco screening alert for (A) all clinics and (B-D) individual clinics. Clinics in (C) and (D) were categorized into three levels based on the number of encounters in which a screening alert was fired during 12 months post alert implementation. Level 1: >1000; level 2: >100 and ≤1000; level 3: ≤100. Line thickness was used to represent these three levels. EHR: electronic health record. M1-M3: medical oncology clinics 1-3. R1-R3: radiation oncology clinics 1-3. S: cancer survivorship clinic.

The screening alert fired in 5121 (2.8% of 180,647) encounters 12 months post implementation. The alert completion rate was 0.55 (2817/5121) based on the staff’s acknowledgment of screening completion in EHRs and 0.32 (1647/5121) based on the completion of EHR documentation of screening results. Both alert completion rates remained stable over time (Figure 2A) but varied considerably across clinics (Figure 2B-D). Among the 2817 encounters where the staff acknowledged completion of screening, 84.7% completed interruptive alerts and 15.4% completed noninterruptive ones.

The support alert was implemented for 5 clinics (medical oncology clinic 2 and 3, radiation oncology clinic 2 and 3, and cancer survivorship clinic) and fired in 1074 encounters. Providers responded without postponing (n=938, 87.3%), discussed tobacco use treatment options (n=640, 59.6%), identified patients who were ready to quit (n=129, 12%), and placed referrals to the cessation clinic (n=22, 2%).

### The Burden of Interruptive Alerts

On average, the number of times a screening alert was fired before completion was 2.7 (range 1.0-12.7 for individual clinics; Table 2); the average number of times a support alert was fired before completion was 2.1 (range 1.8-3.3 for individual clinics; Table 2).

**Table 2:** Alert firing rate of the screening alert and the support alert by clinics.

	Medical oncology^a^			Radiation oncology^b^			Cancer survivorship (S^c^)
	M1^d^	M2	M3	R1^d^	R2	R3	
Screening alert	4.9^e^	1.3	2.2	2.1	1.0	4.0	12.7
Support alert	N/A^f^	1.8	3.3	N/A	2.3	3.0	3.0

^a^M1-M3: medical oncology clinics 1-3.

^b^R1-R3: radiation oncology clinics 1-3.

^c^S: cancer survivorship clinic.

^d^M1 and R1 implemented only the screening alert.

^e^We defined the alert firing rate as the number of times the alert fired during 12 months post alert implementation divided by the number of times the alert was completed during the same period. We did not calculate the alert firing rate at the encounter level because it was undefined (ie, division by 0) for encounters that did not complete the alert.

^f^N/A: not applicable.

On average, time spent completing the screening alert per encounter was 53 seconds (50 seconds for support alert); time spent postponing screening alerts per encounter was 52 seconds (67 seconds for support alerts).

### Factors Associated With Alert Completion

Completion rates of the screening alert and the support alert were balanced across patient subgroups (sex, race, and their interaction).

Among 5121 encounters for which the screening alert was fired, 4425 (86.4%) were “relevant” and 696 (13.6%) were “less relevant” to routine tobacco screening. The alert completion rate for “relevant” encounters was higher than that for “less relevant” ones (2793/4425, 63.1% vs 24/696, 3.5%; *P*<.001).

## Discussion

### Principal Results

We developed and applied EHR activity metrics to monitor two tobacco cessation CDS alerts implemented in 7 cancer clinics. Our metrics were able to capture variation in alert completion across clinics, monitor alert efficacy, identify discrepancies between staff-acknowledged screening completion and screening documentation, and provide insights into the balance between alert efficacy and imposed burden. These findings inform four areas where CDS tool design or use can be improved (Table 3), which we discuss below.

**Table 3:** Key findings from the application of the electronic health record (EHR) activity metrics and implications for clinical decision support (CDS) tool design and use.

Key findings from the application of EHR activity metrics		Implications for CDS tools
**Variation in alert completion**		Potential for improving alert adoption/completion through local adaptation
	The screening alert completion rates varied substantially across the clinics.	Strategies to support use: Use clinic-specific strategies to support CDS tool adoption
	The screening alert completion rate was higher for encounters perceived as relevant to routine tobacco screening by physicians.	Strategies to support use: Consider this factor when promoting the use of tobacco cessation CDS tools among health providers
**Limited alert efficacy**		Potential for improving support alert efficacy
	Providers responded to most support alerts, but few patients were ready to quit, and referral to the tobacco cessation clinic was rare.	Strategies to support use: Use additional strategies (eg, patient education, provider training in patient-provider communication) to increase the impact of the CDS tools
**Inconsistencies between the acknowledgment of alert completion and documented screening**		Potential for improving the accuracy of tracking for alert completion
	EHR documentation of screening results was rare for some clinics, even though their clinic staff acknowledged completion of screening for most encounters.	Design: Improve CDS tool design to allow accurate tracking of screening completion at the alert level Strategies to support use: Use metrics that can accurately track screening completion, such as metrics calculated based on the completion of EHR documentation of screening results
**Interruptive alerts received more responses but also added burden to providers**		Importance of balancing alert efficacy with the burden
	Providers were more responsive to interruptive alerts than noninterruptive ones. Postponing the interruptive alert did not save providers time compared with completing the alert.	Design: Increase the time interval between postponing and refiring an interruptive alert Set a threshold to limit the total number of firing of tobacco cessation alerts during a single encounter

### Improving Alert Adoption and Completion Through Local Adaptation

Clinics varied substantially in completing the alert, calling for clinic-specific strategies to improve alert adoption. We also identified a modifiable factor (ie, the alert encounter relevance) that affects alert completion. Our physician coauthors considered certain encounter types (eg, initial consultation and office visit) to be relevant for routine tobacco use screening, while others (eg, lab visit and radiation oncology treatment visit) were deemed less relevant. While existing guidelines recommend repeating the smoking assessment at every encounter [17,18], we found that the completion rate of the screening alert was much lower for “less relevant” encounters, which may appear to be guideline noncompliance. This finding could be informative for committees that develop tobacco screening and treatment guidelines. Implementation teams that want to enforce the “screening at every encounter” rule may need additional strategies. These could include using provider orientation and local champions to influence the culture surrounding tobacco screening [35].

### Improving Support Alert Efficacy

Although providers responded to support alerts frequently, referral to the tobacco cessation clinic was rare. One reason was that few patients were ready to quit at the point of care. Future programs may incorporate additional strategies, such as patient education, provider training in patient-provider communication, and addressing patient-level barriers (eg, barriers associated with health beliefs and socioeconomic factors) [36,37]. Note that the 2% referral rate may underestimate the effect of the support alert because it was calculated based on referrals directly linked to the alert. If tobacco treatment specialists contacted the patients interested in quitting after the patient visits, these follow-up activities would be documented elsewhere without a link to the alert, or if a patient chose other treatment methods (eg, quitline or medications), the alert-driven referral would not happen.

### Improving Accuracy of Tracking for Alert Completion

The completion rates of the EHR documentation of screening results were lower for some clinics, even though their clinic staff acknowledged screening completion for most encounters. Through discussion with the team coordinating the tobacco cessation program, we identified one major reason for this gap. In clinics using support from patient navigators to complete screening documentation, the clinic staff were likely to bypass the screening but still acknowledged completion. Therefore, measuring EHR documentation is important for the accurate tracking of alert success. We used encounter-level data for this measurement. Alert-level tracking may be necessary for the future development of targeted strategies (eg, provider-specific training) to improve alert adoption. The alert design can be improved to allow this, for example, by disabling the button for acknowledging the completion of screening until the EHR documentation is completed.

### Balancing Alert Efficacy With Burden

Although commonly used, effective integration of e-alerts into the clinical workflow has proven difficult [29-33,38,39]. Medication alerts were frequently overridden by health care providers [29,30,33,40], and providers experienced alert-related burden and fatigue [9,29,31,41]. Our study found that postponing the interruptive alert did not save providers time compared with completing the alert. This was partly due to the refiring of postponed alerts. An overabundance of interruptive alerts in EHRs may lead to frequent “postpone” or “override” actions and user dissatisfaction [31-33]. However, our findings do not support disabling the interruptive alerts, as we found that providers were much more responsive to interruptive alerts than noninterruptive ones. One way to alleviate the alert burden is increasing the time interval between postponing and refiring or setting the maximum number of times (eg, 2 or 3) to fire a tobacco cessation alert during each encounter.

### Contribution to Implementation Science Methods

New methods are needed for monitoring implementation, including automated approaches that reduce the data collection burden [7,42]. We contributed to this literature by developing automatic EHR activity metrics for monitoring the implementation of CDS tools. Our approach has three merits. First, automatic metrics are suitable for rapid periodic evaluation of implementation programs. These metrics can identify deviations and variations of CDS use at clinic and provider levels, which may inform the selection of key informants for interviews to identify causes of deviation and variation, and the development of strategies to improve CDS design and use. Second, EHR activity data work “behind the scenes” to capture EHR use behavior without interruptions [43-45]. Metrics built on this data can reduce reporting bias and may minimize Hawthorne effects (ie, participants’ engagement with an intervention changes when they are aware of attention from observers) [46]. Third, EHRs have been adopted by most US hospitals [47], and EHR-embedded CDS tools are frequently used to support health care quality improvement [1-6]. The ubiquity of EHRs contributes to the generalizability of our approach.

Our work relates to studies using EHR audit logs (one type of EHR activity data) but is different in methodology. The metrics described in these studies measure EHR use and associated burden (eg, total time on EHR, time spent using the EHR after hours, time spent on chart review per patient per day) [48-52] nonspecific to CDS tools. Using EHR audit logs to measure providers’ response to a specific EHR tool is challenging, typically involving manual mapping of low-level actions recorded in the log files to EHR use activities [39,50,53]. We used alert activity data generated by Epic’s built-in functions to eliminate manual mapping.

Prior studies on alert burden focused on medication alerts and used alert override rate and alert volume as markers for burden in the context of de-implementation [30-33,40]. To our knowledge, this study is the first to systematically measure the burden of preventive care alerts. Our findings did not support a simple de-implementation approach but call for better local adaptation to balance alert efficacy and burden.

### Limitations

This study has several limitations. First, the EHR data we analyzed only contained alert-linked referrals to the tobacco cessation clinic. Our analysis may underestimate the actual effect of the support alert. Second, EHR activity data only capture provider interaction with the EHR and lack information about other clinical activities (eg, discussion with patients, pager ringing) during an encounter. In-depth investigations on clinical workflows and their impact on alert response are needed to better understand the variation of alert completion across clinics.

### Conclusions

This study developed EHR activity metrics and demonstrated their use in monitoring the impact of CDS tools implemented by a C3I-funded implementation program that promotes tobacco cessation in patients with cancer. These metrics can be used to guide implementation adaptation and are scalable and adaptable to other settings that use e-alerts to promote adherence to health practice guidelines.

## Appendix

Multimedia Appendix 1 Clinical workflows associated with tobacco cessation alerts.

Multimedia Appendix 2 Electronic health record activity metrics.

Multimedia Appendix 3 Encounter types relevant to tobacco use screening for patients with cancer.

## Abbreviations

BPA: Best Practice Advisory
C3I: Cancer Center Cessation Initiative
CDS: clinical decision support
e-alerts: electronic alerts
EHR: electronic health record
NCI: National Cancer Institute
TCCOE: Tobacco Control Center of Excellence

## Data Availability

Data supporting the study reported in this paper can be made available in deidentified form subject to establishing a data use agreement with the Wake Forest University School of Medicine. The code supporting this study can be accessed on GitHub [54].
