# Supplementary material for: Monitoring the Implementation of Tobacco Cessation Support Tools: Using Novel Electronic Health Record Activity Metrics
Source: JMIR Med Inform. 2023 Mar 2;11:e43097. doi: 10.2196/43097 (PMC10020903; doi:10.2196/43097)
Supplement: Multimedia Appendix 1 [file medinform_v11i1e43097_app1.pdf]

Multimedia Appendix 1. Clinical workflows associated with tobacco cessation alerts

Tobacco-use screening alert

Function: To enhance existing tobacco screening in two ways: (1) support periodic update and completion of EHR documentation on smoking status (including the use of other tobacco products and e-cigarettes) for cancer patients and (2) trigger an EHR prompt (i.e., the Tobacco-use treatment support alert) for providers to provide counseling and/or referral to a cessation clinic for patients who smoke.

Firing condition: Triggered for clinic staff if “current smoker” or “unknown smoking status” was previously documented in the EHR Social History; triggered again three months later if the screening result was positive.

Targets: Clinic staff, including certified medical assistant, certified nurse assistant, licensed nurse, registered medical assistant, registered nurse, and case manager, receive this alert.

Figure A1-1. Screening alert workflow

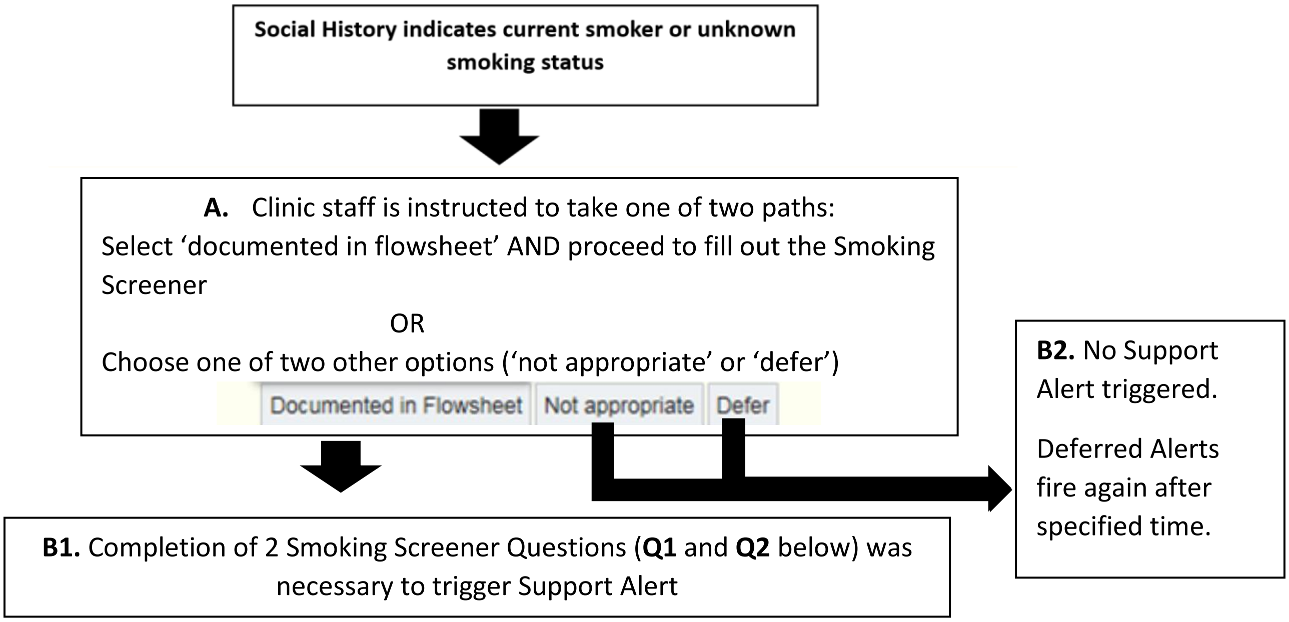

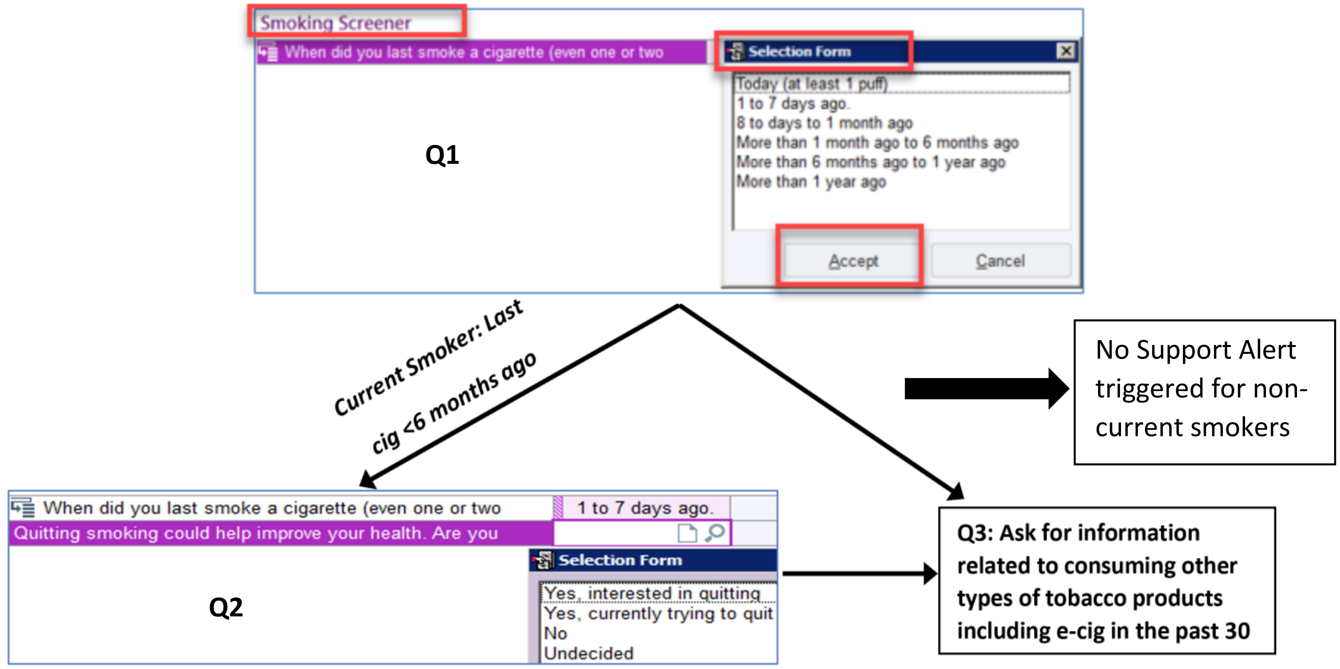

Note:

1. The alert will fire every 3 months for patients with positive screening results (i.e., current smoker, as defined by smoking within the last 6 months).
2. The interruptive version of this alert was designed to fire every 10 minutes if the clinic staff postpones the alert. This time interval can be adjusted based on requests from clinical teams.

Tobacco-use treatment support alert

Function: To prompt the providers to provide counseling and/or referral to a cessation clinic for patients who smoke.

Firing condition: Triggered for clinical provider if the screening result was positive (i.e., current smoker).

Targets: Clinical providers, including physician, physician assistant, nurse practitioner, resident, and physician assistant student, receive this alert.

Figure A1-2. Support alert workflow

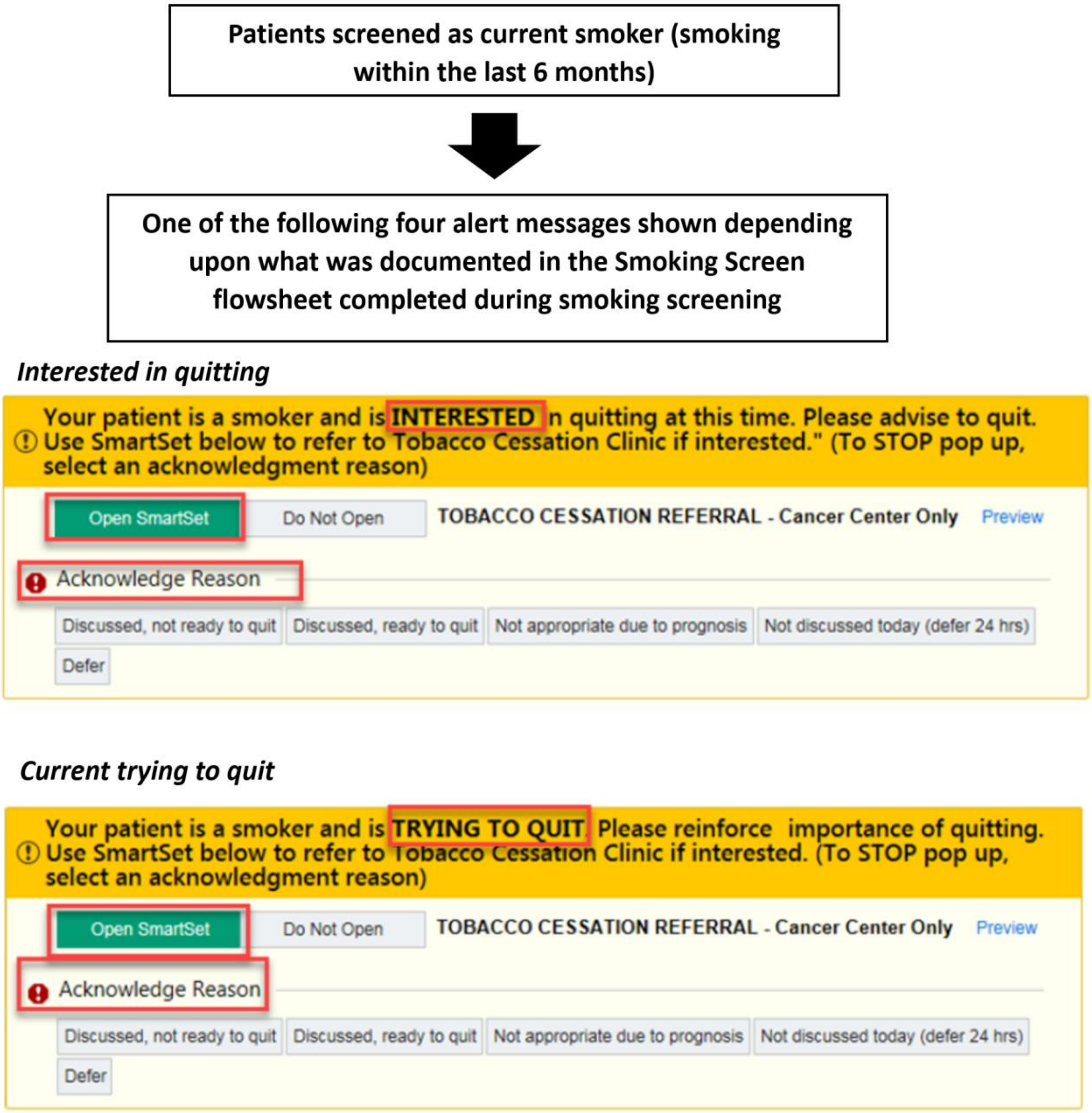

Undecided about quitting

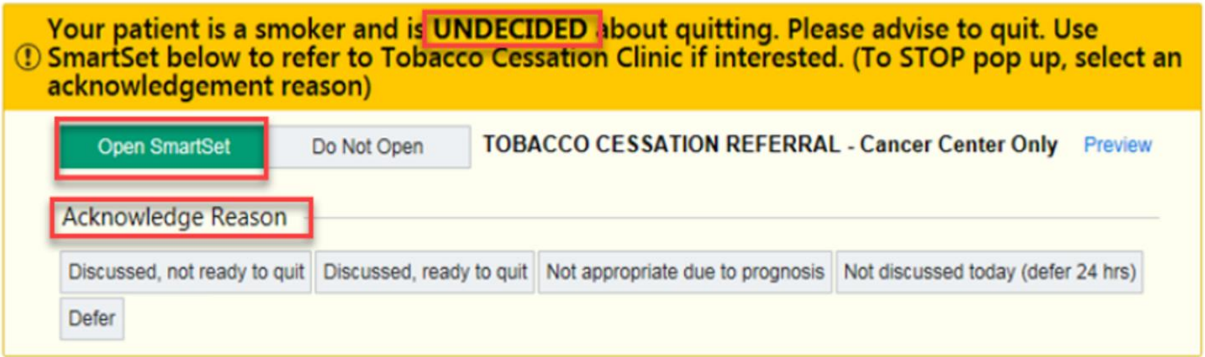

Not interested in quitting

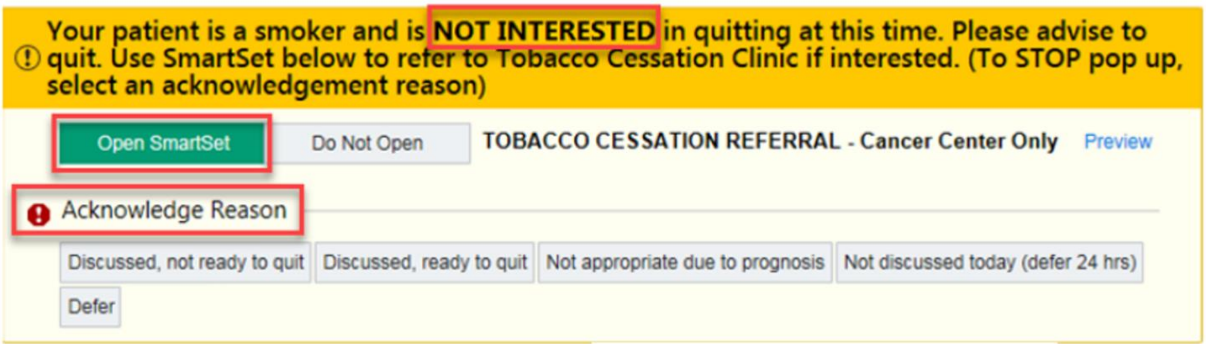

acknowledge reason

If the patient is agreeable to a referral to the Tobacco Cessation Clinic

select Open Smartset

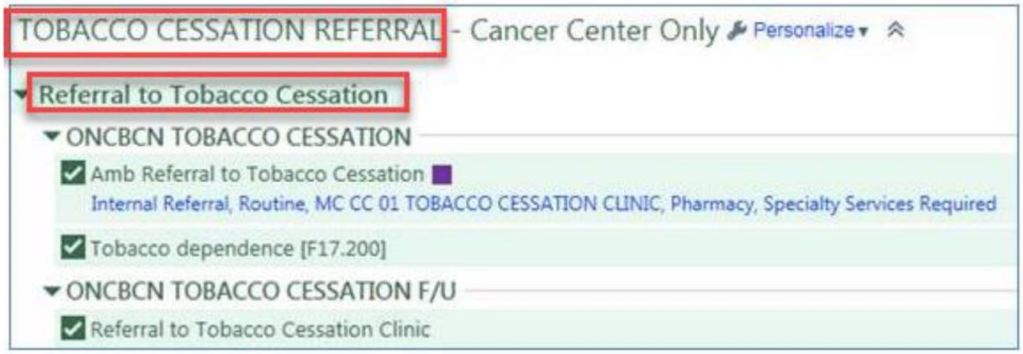

sign the Smartset

Note:

1. In the above figure, the button “Not discussed today (defer 24hrs)” in the “Acknowledge Reason” panel means “the patient declined for counseling or discussion with the provider”.
2. The Open SmartSet button is preselected. Regardless of which alert message window populates, the provider is encouraged to discuss with patient about a referral to the Tobacco Cessation Clinic.
3. The interruptive version of this alert was designed to fire every 10 minutes if the providers postpone the alert. This time interval can be adjusted based on requests from clinical teams.
