# Supplementary material for: Monitoring the Implementation of Tobacco Cessation Support Tools: Using Novel Electronic Health Record Activity Metrics
Source: JMIR Med Inform. 2023 Mar 2;11:e43097. doi: 10.2196/43097 (PMC10020903; doi:10.2196/43097)
Supplement: Multimedia Appendix 2 [file medinform_v11i1e43097_app2.pdf]

## Multimedia Appendix 2. EHR activity metrics

| Metrics | Definition |
|---|---|
| **Completion (combined effects of interruptive and noninterruptive alerts)** | |
| **Alert completion rate** | The number of encounters in which a provider completed the action(s) requested by an alert divided by the number of encounters in which the alert fired during a given time period. |
| | |
| **Burden (interruptive alerts)** | |
| **Alert firing rate** [a] | The number of times an interruptive alert fired during a given time period divided by the number of times the alert was completed during the same period. |
| **Alert handling time** [b] | The average time that providers spent handling (completing or postponing) an interruptive alert per encounter during a given time period. |

[a] We did not calculate alert firing rate at the encounter level, because it was undefined (i.e., division by 0) for encounters that did not complete the alert.

[b] The average time spent completing the alert per encounter was defined as the total time spent completing the alert divided by the total number of encounters in which the alert was completed. The average time spent postponing alerts per encounter was defined as the total time spent postponing the alerts divided by the total number of encounters in which the alert was postponed at least once. The time for handling (completing or postponing) each firing of the alert was calculated as “alert response time point” – “alert firing time point”. The “alert firing time point” records the time point when an alert fired and “alert response time point” records the time point when an action was taken to handle (postpone or complete) the alert. These two variables were extracted from the EHR alert activity table. For the screening alert, “completion” means the staff acknowledged completion of screening in EHR. For the support alert, “completion” means any EHR-recorded action rather than “postponing”, such as discussing with the patient or acknowledging that patient was not appropriate for tobacco-use treatment or declined for counseling (see Figure A1-2 in Multimedia Appendix 1). The section *Methods->EHR Variables Used to Derive the Metrics* in the main paper provides details of EHR alert activity variables.
