# Supplementary material for: Monitoring the Implementation of Tobacco Cessation Support Tools: Using Novel Electronic Health Record Activity Metrics
Source: JMIR Med Inform. 2023 Mar 2;11:e43097. doi: 10.2196/43097 (PMC10020903; doi:10.2196/43097)
Supplement: Multimedia Appendix 3 [file medinform_v11i1e43097_app3.pdf]

Multimedia Appendix 3. Encounter types relevant to tobacco-use screening for cancer patients

Encounter type	Responsible for screening patient smoking status [a]
Clinical Support	X
Erroneous Encounter	X
Evaluation	X
Infusion	X
Initial consult	Yes
Lab visit	X
Nurse Only	Yes
Office Visit	Yes
Orders Only	X
Post-Op	Yes
Procedure visit	Yes
RadOnc On Treatment	X
RadOnc Procedure	X
RadOnc Simulation	X
Return Patient	Yes
Telemedicine	Yes
Transcribe Order	X
Treatment	Yes

[a] Three physicians reviewed and discussed the encounter types to decide which encounter types in principle are appropriate for routine screening for tobacco use in cancer patients.
